# Prospective investigation of complementary and alternative medicine use and subsequent hospitalizations

**DOI:** 10.1186/1472-6882-8-19

**Published:** 2008-05-08

**Authors:** Tyler C Smith, Besa Smith, Margaret AK Ryan

**Affiliations:** 1 Department of Defense Center for Deployment Health Research, Naval Health Research Center, 140 Sylvester Road, San Diego, CA 92106, USA

## Abstract

**Background:**

The prevalence of complementary and alternative medicine (CAM) use has been estimated to be as high as 65% in some populations. However, there has been little objective research into the possible risks or benefits of unmanaged CAM therapies.

**Methods:**

In this prospective study of active duty US Navy and Marine Corps personnel, the association between self-reported practitioner-assisted or self-administered CAM use and future hospitalization was investigated. Cox regression models were used to examine risk of hospitalization due to any cause over the follow-up period from date of questionnaire submission, until hospitalization, separation from the military, or end of observation period (June 30, 2004), whichever occurred first.

**Results:**

After adjusting for baseline health, baseline trust and satisfaction with conventional medicine, and demographic characteristics, those who reported self-administering two or more CAM therapies were significantly less likely to be hospitalized for any cause when compared with those who did not self-administer CAM (HR = 0.38; 95% CI = 0.17, 0.86). Use of multiple practitioner-assisted CAM was not associated with a significant decrease or increase of risk for future hospitalization (HR = 1.86; 95 percent confidence interval = 0.96-3.63).

**Conclusion:**

While there were limitations to these analyses, this investigation utilized an objective measure of health to investigate the potential health effects of CAM therapies and found a modest reduction in the overall risk of hospitalization associated with self-administration of two or more CAM therapies. In contrast, use of practitioner-assisted CAM was not associated with a protective effect.

## Background

Complementary and alternative medicine (CAM) therapies have been used for centuries in one form or another, with deep roots in many ancient cultures [1,2]. Numerous studies have reported prevalence of CAM use to be from 9% to 65% in the United States, Canada, Australia, Denmark, and the United Kingdom [3-12]. Variations in the reported prevalence of CAM use likely come from descriptions and classification of CAM [4]. CAM has been described as a diagnosis, treatment, and/or prevention that complements mainstream medicine by contributing to a common whole, satisfying demand not met by orthodoxy or diversifying the conceptual frameworks of medicine [3]. With annual out-of-pocket expenditures related to CAM use in the United States estimated at more than $27 billion in 1997 [5], the prevalence of CAM use highlights its significance in the current healthcare environment.

Investigations of CAM use in US adult populations have suggested that women, those of white race/ethnicity, and those with higher education levels are more likely to use CAM therapies [5,7,13]. Investigations of US military personnel have also suggested that those who use CAM are more likely to be officers, in technical support occupations, report more sick days, and report more bodily pain [7].

CAM is often used as an alternative or complement to conventional medicine practices for prevention and/or treatment of disease. Among the most commonly reported CAM therapies are herbal medicine, massage, high-dose megavitamins, and relaxation therapies [5,7,13-17]. Uses range from daily vitamin or herbal supplementation to ward off common colds, to therapies for symptom reduction of chronic illnesses such as fibromyalgia [18] or rheumatic diseases [19], for slowing or treating cancers [20-23], and for relief of symptoms from other serious diseases such as AIDS [24,25] or diabetes [26].

There has been little research, however, into the possible risks of unmanaged therapies [27-29] as well as the benefits CAM use may offer in protection against disease or illness [30]. The objective of this study was to prospectively investigate the association of CAM use with subsequent hospitalization in a healthy, active-duty US military population with equal access to conventional medical care.

## Methods

### Study population

In December 2000, a random sample of 5,000 US active duty and Reserve Navy and Marine Corps personnel was drawn from military rosters of approximately 550,000. Prospective participants were sent a questionnaire to assess baseline health and use of CAM therapies [7]. There were 1,446 who responded out of 3,683 who received a study questionnaire and were eligible to participate. Among the ineligible subjects, 16 did not meet the initial enrollment criteria and 1,301 could not be located. Of the 3,683 eligible subjects, 49 refused to participate, 2,188 did not respond after repeated mailings, and 1,446 responded. This resulted in a response rate of 39.3%. Some proportional differences existed between responders and nonresponders when considering gender, age, marital status, rank, service branch, and occupational category. Respondents were slightly different than the target population of US Navy and Marine Corps, and tended to be older, married, officers, in the Navy, and in the field of healthcare [7]. Reserve personnel (n = 74) were excluded from these analyses due to differential access to Department of Defense (DoD) healthcare, leaving 1,372 respondents included in this analysis.

This research has been conducted in compliance with all applicable federal regulations governing the protection of human subjects in research (Protocol NHRC.2001.0001).

### Questionnaire data

The 10-page survey instrument was developed by the study team to ascertain the use of CAM therapies, feelings of general health, and some demographic characteristics, while taking no more than 30 minutes to complete. Each invited participant was mailed up to 3 surveys with prepaid return envelopes, based on a modified version of the Dilman method [31]. Self-reported data included race/ethnicity (white, Hispanic, black, Asian or Pacific Islander, other), and salary (<$19,999; $20,000–34,999; $35,000–49,999; $50,000–74,999; ≥ $75,000). Also included in the questionnaire were questions to assess general health, bodily pain, medical care, and 24 questions regarding health conditions in the previous 12 months (lung problems, pneumonia, respiratory infections; high blood pressure; heart problems; diabetes; tumors or cancers; digestive problems; urinary tract problems; gynecologic or menstrual problems; neurological problems; sprains or muscle strains; skin or dermatological problems; allergies; dizziness; anxiety; depression; insomnia or trouble falling asleep; addictive problems with alcohol or drugs; obesity; chronic dental problems; arthritis or rheumatism; back problems; severe headaches; any other kind of chronic pain; and chronic fatigue syndrome). Health conditions reported in the previous 12 months were categorized into 0–1, 2–3, 4–5, or 6 or more.

#### CAM data

Included in the questionnaire data were 13 practitioner-assisted CAM therapies in the previous 12 months (acupuncture, chiropractic services, homeopathy, spiritual/religious healing, energy healing, folk remedies, massage therapies, self-help group, biofeedback, hypnosis, exercise/movement therapy, psychotherapy, and art/music therapy; categorized into 0, 1, or 2 or more), 8 self-administered CAM uses in the previous 12 months (high-dose mega-vitamin, herbal therapy, homeopathy, prayer/spiritual practice, energy healing, folk remedies, relaxation techniques, and aromatherapy; categorized into 0, 1, or 2 or more),

### Demographic data

Demographic data were provided by the Defense Manpower Data Center, Monterey Bay, California. These data included sex, age (categorized by approximate quartile age groups: 18 to 24 years, 25 to 31 years, 32 to 38 years, and 39 to 57 years), rank (categorized into enlisted and officer), marital status (married or not), service branch (Navy and Marine Corps), DoD primary occupational specialties (10 major categories, defined by the DoD *Occupational Conversion Manual*) [32], highest level of education (some high school or diploma, some college, and college degree), and date of separation from military service.

### Hospitalization data

Hospitalization data for each service member included, if applicable, date of admission and up to 8 discharge diagnoses coded using the *International Classification of Diseases, 9th Revision, Clinical Modification *(ICD-9-CM) [33]. Data were captured from all DoD military treatment facilities as well as any private hospitals billing DoD for care during the period of December 1, 2000, through June 30, 2004. Hospitalizations were scanned in chronological order, and diagnostic fields were scanned in numeric order for the diagnostic codes of interest. Probability of hospitalization for "any cause" was examined using time until first hospitalization during the time period of survey submission until June 30, 2004. Hospitalization data indicating a diagnosis in the broad diagnostic category of complications of pregnancy, childbirth, and puerperium were not included.

### Statistical analyses

Univariate analyses were performed to assess the significance of associations between hospitalization, demographic, and baseline self-reported variables. An exploratory analysis was developed to further assess variables of interest for significant associations and possible confounding, while simultaneously adjusting for all other variables in the model. Using regression diagnostics, collinearity among variables was assessed.

Cox's proportional hazards time-to-event modeling was used to independently compare (1) the hospitalization experience of practitioner-assisted CAM users with non practitioner-assisted CAM users and (2) the hospitalization experience of self-administered CAM users with the hospitalization experience of non self-administered CAM users, while accounting for attrition from active-duty service over the follow-up period [34]. Follow-up time was calculated from date of enrollment until hospitalization, separation from active-duty service, or June 30, 2004, whichever occurred first. The saturated Cox regression model was reduced by a manual, backward, stepwise elimination approach to investigate confounding, removing those variables that were not independently associated at an alpha cutoff level of 0.05 and not determined to be confounders. Adjusted hazard ratios (HRs) and 95% confidence intervals (CIs) were calculated for the hazard of hospitalization. Additionally, the cumulative probability of hospitalization as a function of time was graphed after stratification by different levels of CAM use. All data management and analyses were completed using SAS, version 9.1.2 (SAS Institute, Inc., Cary, North Carolina).

## Results

Data for this analysis were complete and available for 1,319 of the 1,372 active-duty personnel who had elected to participate and submitted their questionnaire (96.1%). There were 434 (32.9%) who reported practitioner-assisted CAM therapies and 427 (32.4%) who reported self-administered CAM therapies in the 12 months prior to taking the survey (Table 1). Although the prevalence of those reporting practitioner-assisted and self-administered CAM use was similar, those using practitioner-assisted CAM included only half of those self-administering CAM (Table 1). As shown in Table 1, 55% of the study population reported fewer than 4 conditions in the previous year, 67% reported very good or excellent health, 56% reported none or very mild bodily pain in the past 4 weeks, 45% reported being very satisfied with their conventional medicine doctor, and 50% reported trusting their conventional medicine doctor completely or a lot. Demographic and military characteristics indicated that 78% of the study population were male, 50% were aged 18–31 years, 60% were married, 66% were white, 50% had some college education, 52% earned $35,000 or more each year, 82% were enlisted personnel, 77% were Navy, and 22% were combat specialists.

**Table 1:** Population characteristics by practitioner-assisted or self-administered CAM therapies

**Characteristic**	**Population** N = 1,319		**Practitioner-assisted CAM** n = 434 (32.9%)		**Self-administered CAM** n = 427 (32.4%)	
	
	Number	%	Number	%	Number	%
Practitioner-assisted CAM						
0 therapies	885	67.1	--	--	192	45.0
1 therapy	254	19.2	--	--	102	23.9
≥ 2 therapies	180	13.7	--	--	133	31.2
Self-administered CAM						
0 therapies	892	67.6	199	45.8	--	--
1 therapy	237	18.0	114	26.3	--	--
≥ 2 therapies	190	14.4	121	27.9	--	--
Health conditions in past 12 months						
0–1 conditions	216	16.4	29	6.7	45	10.5
2–3 conditions	507	38.4	145	33.4	145	34.0
4–5 conditions	327	24.8	127	29.3	112	26.2
≥ 6 conditions	269	20.4	133	30.6	125	29.3
Bodily pain in past 4 weeks						
None/very mild	742	56.2	174	40.1	228	53.4
Mild	308	23.4	122	28.1	89	20.8
Moderate/severe	269	20.4	138	31.8	110	25.8
Satisfaction with conventional medical doctor						
Not at all or not very much	196	14.9	77	17.7	73	17.1
Somewhat	528	40.0	187	43.1	185	43.3
Very	595	45.1	170	39.2	169	39.6
Level of trust in conventional medical doctor						
Little or not at all	238	18.0	77	17.7	88	20.6
Some	423	32.0	140	32.3	130	30.4
Completely or a lot	658	49.9	217	50.0	209	49.0
General health						
Poor or fair	65	4.9	26	6.0	27	6.3
Good	367	27.8	146	33.6	108	25.3
Very good or excellent	887	67.3	262	60.4	292	68.4
Sex						
Male	1,029	78.0	323	74.4	301	70.5
Female	290	22.0	111	25.6	126	29.5
Age (years)						
18–24	305	23.1	102	23.5	108	25.3
25–31	348	26.4	114	26.3	102	23.9
32–38	342	25.9	113	26.0	111	26.0
39–57	324	24.6	105	24.2	106	24.8
Marital status						
Single or divorced	528	40.0	199	45.9	183	42.9
Married	791	60.0	235	54.1	244	57.1
Race/ethnicity						
White	866	65.7	303	69.8	293	68.6
Hispanic	123	9.3	28	6.5	37	8.7
Black	190	14.4	59	13.6	54	12.7
Asian and Pacific Islander	92	7.0	31	7.1	27	6.3
Other	48	3.6	13	3.0	16	3.7
Education level						
Some high school or diploma	367	27.8	115	26.5	102	23.9
Some college	654	49.6	226	52.1	221	51.8
College degree	298	22.6	93	21.4	104	24.3
Salary						
≤ $19,999	260	19.7	83	19.1	94	22.0
$20,000-$34,999	377	28.6	123	28.3	111	26.0
$35,000-$49,999	285	21.6	100	23.1	92	21.6
$50,000-$74,999	239	18.1	79	18.2	73	17.1
≥ $75,000	158	12.0	49	11.3	57	13.3
Rank						
Enlisted	1,082	82.0	362	83.4	339	79.4
Officer	237	18.0	72	16.6	88	20.6
Service						
Regular Navy	1,017	77.1	333	76.7	341	79.9
Regular Marine Corps	302	22.9	101	23.3	86	20.1
Occupational category						
Combat specialists	287	21.8	89	20.5	103	24.1
Electronic equipment repair	192	14.6	63	14.5	56	13.1
Communications/intelligence	97	7.4	37	8.5	32	7.5
Healthcare	115	8.7	38	8.8	44	10.3
Functional support	220	16.7	70	16.1	60	14.1
Electrical/mechanical repair	236	17.9	71	16.4	72	16.9
Craft workers	33	2.5	17	3.9	11	2.6
Service and supply handlers	82	6.2	24	5.5	27	6.3
Students, trainees, other	57	4.3	25	5.8	22	5.2

Those reporting practitioner-assisted CAM therapy in the previous 12 months were proportionally more likely to be female, enlisted, single or divorced, white, with some college education, report 4 or more health problems, good general health, and moderate to severe bodily pain. With some variations, those reporting self-administering at least 1 CAM therapy in the previous 12 months were proportionally more likely to be female, officers, single or divorced, combat specialists, white, with a college degree, report 6 or more health conditions, and report moderate to severe bodily pain (Table 1).

Regression diagnostics for investigation of the pairwise correlations and the variance inflation factor suggested no discernable collinearity among the variables, although salary and rank were noted as moderately correlated. The proportional hazards assumption was assured by visual inspection of cumulative distribution function plots and tested by the addition to the model of interaction terms between the main variables and time. There were no statistically significant time-by-variable interactions observed using a p-value cutoff of 0.10.

After adjustment for potential confounders, reporting 1 or reporting 2 or more practitioner-assisted CAM therapy was not associated with any-cause hospitalization (Table 2). After adjustment for potential confounders in the Cox proportional hazards regression, those self-administering 1 CAM therapy appeared potentially protected (HR = 0.62; 95% CI, 0.33–1.17) and those self-administering 2 or more CAM therapies were significantly protected (HR = 0.38; 95% CI, 0.17–0.86) from any-cause hospitalization, in comparison with those who did not report self-administering CAM therapies (Table 2).

**Table 2:** Adjusted hazard ratios for any-cause hospitalization in personnel reporting practitioner-assisted or self-administered CAM therapies

**Characteristic**	**Population**		**Hospitalized**			
	Number	%	Number	%	HR*	95% CI
Practitioner-assisted CAM						
0 therapies^†^	885	67.1	57	66.3	1.00	--
1 therapy	254	19.3	14	16.3	0.99	0.53, 1.84
≥ 2 therapies	180	13.6	15	17.4	1.86	0.96, 3.63
Self-administered CAM						
0 therapies^†^	892	67.6	65	75.6	1.00	--
1 therapy	237	18.0	13	15.1	0.62	0.33, 1.17
≥ 2 therapies	190	14.4	8	9.3	0.38	0.17, 0.86
Health conditions in past 12 months						
0–1 conditions^†^	216	16.4	11	12.8	1.00	--
2–3 conditions	507	38.4	26	30.2	1.10	0.53, 2.28
4–5 conditions	327	24.8	22	26.7	1.46	0.68, 3.18
≥ 6 conditions	269	20.4	27	31.4	2.22	0.99, 4.98
Bodily pain in past 4 weeks						
None/very mild^†^	742	56.2	45	52.3	1.00	--
Mild	308	23.4	22	25.6	1.02	0.58, 1.79
Moderate/severe	269	20.4	19	22.1	0.94	0.50, 1.77
Level of satisfaction with conventional medical doctor						
Not at all or not very^†^	196	14.9	10	11.6	1.00	--
Somewhat	528	40.0	32	37.2	1.78	0.79, 4.02
Very	595	45.1	44	51.2	2.30	0.91, 5.82
Level of trust in conventional medical doctor						
Little or not at all^†^	238	18.0	15	17.4	1.00	--
Some	423	32.0	27	31.4	0.80	0.39, 1.66
Completely or a lot	658	49.9	44	51.2	0.69	0.31, 1.53
General health						
Poor or fair^†^	65	4.9	8	9.3	1.00	--
Good	367	27.8	27	31.4	0.45	0.20, 1.03
Very good or excellent	887	67.3	51	59.3	0.39	0.17, 0.90
Sex						
Male^†^	1,029	78.0	63	73.3	1.00	--
Female	290	22.0	23	26.7	1.25	0.72, 2.18
Age (years)						
18–24^†^	305	23.1	18	20.9	1.00	--
25–31	348	26.4	17	19.8	0.64	0.30, 1.36
32–38	342	25.9	24	27.9	1.15	0.53, 2.48
39–57	324	24.6	27	31.4	1.17	0.51, 2.70
Marital status						
Single or divorced^†^	528	40.0	27	31.4	1.00	--
Married	791	60.0	59	68.6	1.68	0.99, 2.86
Race/ethnicity						
White^†^	866	65.7	54	62.8	1.00	--
Hispanic	123	9.3	11	12.8	1.61	0.81, 3.20
Black	190	14.4	11	12.8	0.90	0.46, 1.79
Asian and Pacific Islander	92	7.0	6	7.0	0.77	0.32, 1.87
Other	48	3.6	4	4.7	1.20	0.42, 3.48
Education level						
Some high school or diploma^†^	367	27.8	25	29.1	1.00	--
Some college	654	49.6	37	43.0	0.85	0.49, 1.46
College degree	298	22.6	24	27.9	1.44	0.63, 3.32
Salary						
≤ $19,999^†^	260	19.7	11	12.8	1.00	--
$20,000-$34,999	377	28.6	35	40.7	1.92	0.87, 4.22
$35,000-$49,999	285	21.6	15	17.4	1.01	0.38, 2.64
$50,000-$74,999	239	18.1	12	14.0	0.68	0.24, 1.95
≥ $75,000	158	12.0	13	15.1	1.01	0.30, 3.47
Rank						
Enlisted^†^	1,082	82.0	70	81.4	1.00	--
Officer	237	18.0	16	18.6	1.02	0.40, 2.60
Service						
Regular Navy^†^	1,017	77.1	69	80.2	1.00	--
Regular Marine Corps	302	22.9	17	19.8	1.17	0.64, 2.13
Occupational category						
Combat specialists^†^	287	21.8	15	17.4	1.00	--
Electronic equipment repair	192	14.6	7	8.1	0.73	0.29, 1.85
Communications/intelligence	97	7.4	5	5.8	1.17	0.41, 3.37
Healthcare	115	8.7	16	18.6	2.63	1.21, 5.72
Functional support	220	16.7	18	20.9	1.60	0.76, 3.35
Electrical/mechanical repair	236	17.9	19	22.1	1.60	0.76, 3.36
Craft workers	33	2.5	1	1.2	0.44	0.06, 3.55
Service and supply handlers	82	6.2	2	2.3	0.52	0.12, 2.30
Students, trainees, other	57	4.3	3	3.5	1.11	0.31, 3.99

*HR = adjusted hazards ratio; CI = 95% confidence interval.

^†^Reference category.

Table 2 also shows other independent risk factors for hospitalization. Those reporting very good or excellent health were at 0.39 times the risk of hospitalization when compared with those reporting poor or fair health (95% CI, 0.17–0.90). Healthcare workers were at 2.63 times the risk of hospitalization in comparison with combat specialists (95% CI, 1.21–5.72).

**Figure 1:**
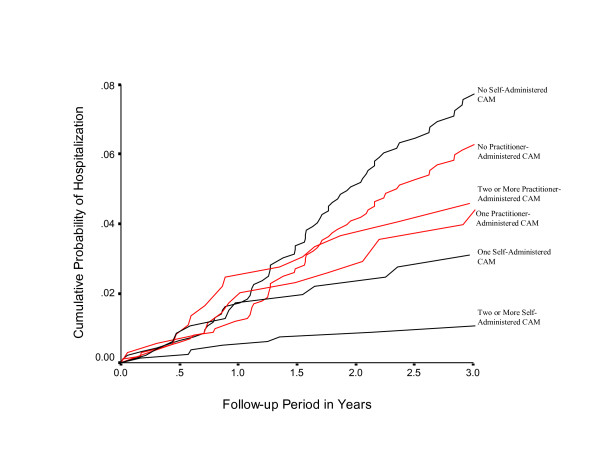
Cumulative probability of any-cause hospitalization in Department of Defense hospitals from date of questionnaire submission until June 30, 2004, by practitioner-assisted and self-administered CAM therapy.

Figure 1 shows the cumulative probability of hospitalization based on practitioner-assisted and self-administered CAM use, while simultaneously adjusting for all other variables in the model. The statistically significant difference associated with 2 or more self-administered CAM therapies (lowest probability of hospitalization) is apparent when compared with the curve of the highest probability of hospitalization among those not self-administering any CAM therapy. Although the step functions showed some sign of instability over the follow-up period, there was no statistically significant sign (p value < 0.10) of temporal bias within the two groups of CAM users.

Investigation of the 13 specific practitioner-assisted and the 8 self-administered CAM therapies identified only two statistically significant predictors of hospitalization (Table 3). After adjustment for potential confounders in the Cox proportional hazards regression, those who were assisted by a practitioner with acupuncture (HR, 3.92; 95% CI, 1.53–10.06) or chiropractic services (HR = 1.96; 95% CI, 1.01–3.80) were at increased risk for future hospitalization in comparison with those not self-reporting such CAM use. Investigation of self-administered CAM uses found no statistically significant associations, but did find many of the therapies to tend toward a protective effect. Those self-administering high-dose mega-vitamins, herbal therapies, prayer or spiritual practices, and aromatherapies, while statistically insignificant individually, likely added to the overall significant protective effect seen in Table 1.

**Table 3:** Adjusted hazard for any-cause hospitalization in personnel reporting specific practitioner-assisted and self-administered CAM uses in the last 12 months

**CAM characteristic**	**CAM use**		**Hospitalized**			
	Number	%	Number	%	HR*	95% CI
Practitioner-assisted CAM						
Exercise/movement therapy	245	18.6	17	19.8	1.13	0.64, 1.97
Massage therapies	181	13.7	12	14.0	0.98	0.51, 1.86
Chiropractic services	113	8.6	11	12.8	1.96	1.01, 3.80
Art or music therapy	41	3.1	0	0.0	--	--
Acupuncture	32	2.4	5	5.8	3.92	1.53, 10.06
Spiritual/religious healing	30	2.3	2	2.3	1.14	0.27, 4.81
Folk remedies	30	2.3	3	3.5	1.52	0.46, 5.09
Energy healing	24	1.8	3	3.5	2.12	0.63, 7.17
Psychotherapy	18	1.4	3	3.5	1.57	0.46, 5.41
Homeopathy	17	1.3	1	1.2	--	--
Self-help group	17	1.3	0	0.0	--	--
Hypnosis	11	0.8	0	0.0	--	--
Biofeedback	9	0.7	0	0.0	--	--
						
Self-administered CAM						
Herbal therapy	208	15.8	9	10.5	0.62	0.30, 1.26
High-dose or mega-vitamin therapy	148	11.2	5	5.8	0.42	0.17, 1.06
Relaxation techniques	139	10.5	9	10.5	1.97	0.47, 1.99
Prayer or spiritual practice	132	10.0	4	4.7	0.39	0.14, 1.07
Aromatherapy	68	5.2	2	2.3	0.37	0.09, 1.53
Energy healing	34	2.6	3	3.5	1.28	0.39, 4.19
Folk remedies	32	2.4	0	0.0	--	--
Homeopathy	18	1.4	0	0.0	--	--

*HR = adjusted hazards ratio; CI = 95% confidence interval.

## Discussion

The increasing trend of complementary and alternative medicine (CAM) use is likely to continue as physicians and patients search for new therapies to improve quality of life or identify alternative and less-toxic forms of therapy while remaining congruent with personal values and beliefs [5,16,35,36]. As the prevalence of CAM use grows however, so does concern over possible side effects from misuse, abuse, and interactions of unmanaged therapies [27-30]. Since there is little known about the risks and benefits of CAM use, and more than 1 in 3 US Navy and Marine Corps personnel use some form of CAM [7], this investigation sought to prospectively document the association of CAM use and subsequent hospitalization in a healthy active-duty population. These data suggest that practitioner-assisted CAM use was marginally associated with an increased risk of future hospitalization, while use of self-administered CAM therapies was significantly associated with a decrease in risk of future hospitalization.

Although not statistically significant, elevated hospitalization risk associated with practitioner-assisted CAM merits consideration. Although these analyses controlled for differences in self-reported general health, bodily pain, and health conditions, this elevated risk may be reflective of seeking healthcare from a professional for a longstanding condition for which conventional medicine has not provided a solution. This explanation is supported by the investigation of specific CAM treatments (Table 3). Only those who received practitioner-assisted acupuncture and chiropractic therapy were at increased risk of hospitalization, suggesting that these CAM users may have had conditions characterized by chronic pain, necessitating increased uses of both CAM and conventional medicine. However, investigation of diagnostic codes did not reveal specific trends of illness. More study into the temporal sequence of CAM and conventional medicine use among those with chronic pain conditions is likely worthwhile.

The finding that those self-administering a single CAM therapy were somewhat protected from future hospitalization, and those self-administering 2 or more CAM therapies were statistically significantly protected, is interesting. This may reflect an elevated individual role and interest in personal health, leading to more involvement in one's own self-care. This explanation is supported by investigation of the specific CAM uses. Table 3 shows that no single self-administered CAM use was statistically significant, but high-dose mega-vitamins, prayer or spiritual practice, and aromatherapy all had more than a suggested twofold protective effect against hospitalization. Another possible explanation of the observed health of CAM users may be the placebo affect [37-39]. The application of hospitalization, as a relatively objective measure of severe health problems, makes this explanation much less likely. Whether the apparent decrease in risk for adverse health outcomes is due to a healthy mindset and hypervigilance towards one's own health or a direct effect of a certain therapy should be considered for future study using a more controlled analytic design.

This study had important limitations that should be noted. First, we selected a broad definition of CAM based on our available survey data. CAM may be defined differently in other research, especially since CAM is, by its nature, somewhat dynamic and evolving. The CAM treatments "psychotherapy" and "self-help groups" are among the more contested inclusions in any definition of CAM. Please note, however, that fewer than 2% of our sample reported use of these treatments. Exclusion of psychotherapy and self-help groups from the CAM definition did not change results of this analysis. Still, these analyses should be interpreted with the various CAM definitions in mind. Approximately 40% of the Navy and Marine Corps population contacted elected to participate, diminishing the ability to generalize these findings to all Navy and Marine Corps personnel or the US military in general. These data were self-reported, and recall bias may be problematic when trying to identify and quantify CAM use. Further, uncertainty caused by labels given to CAM therapies that may not explicitly describe the varying forms of the therapy within the label may cause over or under-reporting of a therapy. Additionally, these data may lack the power to identify significant results with modest levels of association. Conversely, because these analyses were not mathematically adjusted for multiple comparisons, some findings may simply be due to chance. Furthermore, it was not possible to determine if differences in CAM use occurred from the time between survey completion and end of follow-up for hospitalization surveillance. The average time from survey submission to hospitalization was over one year and only CAM use at the time of the survey completion was able to be assessed. Lastly, this investigation focused on morbidity severe enough to require hospitalization, not a broader spectrum of health outcomes.

Despite these limitations, these analyses offer the first exploratory, population-based epidemiologic investigation of a diverse set of CAM therapies that may be of benefit or risk of morbidity among CAM users and nonusers. Hospitalizations are an objective outcome measure, in contrast to self-reported symptoms or illnesses. These hospitalization data are very complete because active-duty military personnel have ready access to essentially free medical care in DoD facilities and have medical care coverage in private hospitals for emergencies. Since active-duty personnel seldom seek care outside the DoD healthcare system, it is likely that these data captured virtually 100% of the most serious health outcomes. Lastly, the use of sophisticated statistical modeling techniques with many variables to adjust for possible confounding, and the integration of data from diverse sources, allowed for the quantification of hospitalization risk over time associated with CAM use.

## Conclusion

This analysis prospectively quantified reported CAM use in relation to adverse health outcomes requiring hospitalization. Using an objective outcome measure in a well-defined population of young adults, these data suggest a statistically significant protective effect associated with self-administrated CAM therapies. In contrast, use of practitioner-assisted CAM therapies was not associated with decreased rates of subsequent hospitalization. More rigorous testing of CAM therapies and interactions with conventional therapies is possible and should be conducted.

## Competing interests

The authors declare that they have no competing interests.

## Authors' contributions

TS and BS performed the statistical analysis. All authors helped conceive the study, participated in its design and coordination, and helped to draft the manuscript. All authors read and approved the final manuscript.

## Pre-publication history

The pre-publication history for this paper can be accessed here:


